# Discogenic Low Back Pain: Anatomy, Pathophysiology and Treatments of Intervertebral Disc Degeneration

**DOI:** 10.3390/ijms24010208

**Published:** 2022-12-22

**Authors:** Isma Liza Mohd Isa, Seong Lin Teoh, Nurul Huda Mohd Nor, Sabarul Afian Mokhtar

**Affiliations:** 1 Department of Anatomy, Faculty of Medicine, Universiti Kebangsaan Malaysia, Kuala Lumpur 56000, Malaysia; 2 SFI Research Centre for Medical Devices, University of Galway, H91W2TY Galway, Ireland; 3 Department of Human Anatomy, Faculty of Medicine and Health Sciences, Universiti Putra Malaysia, Selangor 43400, Malaysia; 4 Department of Orthopaedics and Traumatology, Faculty of Medicine, Universiti Kebangsaan Malaysia, Kuala Lumpur 56000, Malaysia

**Keywords:** intervertebral disc degeneration, discogenic low back pain, inflammation, innervation, pain mechanisms

## Abstract

Intervertebral disc (IVD) degeneration is a major contributing factor for discogenic low back pain (LBP), causing a significant global disability. The IVD consists of an inner core proteoglycan-rich nucleus pulposus (NP) and outer lamellae collagen-rich annulus fibrosus (AF) and is confined by a cartilage end plate (CEP), providing structural support and shock absorption against mechanical loads. Changes to degenerative cascades in the IVD cause dysfunction and instability in the lumbar spine. Various treatments include pharmacological, rehabilitation or surgical interventions that aim to relieve pain; however, these modalities do not halt the pathologic events of disc degeneration or promote tissue regeneration. Loss of stem and progenitor markers, imbalance of the extracellular matrix (ECM), increase of inflammation, sensory hyperinnervation and vascularization, and associated signaling pathways have been identified as the onset and progression of disc degeneration. To better understand the pain originating from IVD, our review focuses on the anatomy of IVD and the pathophysiology of disc degeneration that contribute to the development of discogenic pain. We highlight the key mechanisms and associated signaling pathways underlying disc degeneration causing discogenic back pain, current clinical treatments, clinical perspective and directions of future therapies. Our review comprehensively provides a better understanding of healthy IVD and degenerative events of the IVD associated with discogenic pain, which helps to model painful disc degeneration as a therapeutic platform and to identify signaling pathways as therapeutic targets for the future treatment of discogenic pain.

## 1. Introduction

A global burden of disease study reported that low back pain (LBP) is a significant cause of disability-adjusted life-years (DALYs) worldwide [1]. The prevalence of people with LBP was reportedly 377.5 million at any point in 1990 and increased to 577.0 million in 2017 [2]. In Malaysia, approximately 50.2% of musculoskeletal disorder cases were commonly reported on the lumbar spine [3]. The annual healthcare expenditures of LBP were estimated at 19.77 billion GBP in the United Kingdom [4] and 100 billion USD in the United States [5]. For the Asian region, the estimated economic cost of work-related LBP was 1.2 trillion JPY in Japan [6], indicating an enormous economic burden on society.

LBP is a painful condition related to the musculoskeletal system. Most back pain resolves independently, without active intervention; however, some individuals suffer acute and severe LBP that may progress to chronic LBP [7]. The etiology of LBP is multifactorial, but intervertebral disc (IVD) degeneration is considered one of the primary causes of LBP, accounting for around 26–42% of patients with LBP [8]. The IVD is a fibrocartilaginous tissue that connects adjacent vertebrae, which is composed of an inner core proteoglycan-rich nucleus pulposus (NP), outer region collagen-rich annulus fibrosus (AF) and confined by cartilaginous endplate (CEP) [9]. The IVD provides mechanical stability to the spine, allows movement at the level of the motion segment, and preserves the correct spatial alignment of the vertebrae and facet joints [10]. Degeneration of IVD starts in young adults and progresses with age and ongoing degeneration under pathological insults. It is associated with a reduced capacity for intrinsic self-repair in the tissue, including a decrease in NP progenitor cells [11]. Ageing has been linked to increased cellular senescence, which modifies cellular phenotype and extracellular matrix (ECM) functions. Dysregulation of ECM content is correlated with early-stage disc degeneration [12], leading to inflammation. The onset of discogenic pain has been associated with increased inflammation, inducing nociceptive nerve ingrowth into the aneural disc, which contributes to the development of pain [13]. Other factors that contribute to disc degeneration include mechanical load and injury, low nutrition supply [14], genetics [15], smoking [16] and obesity [17].

Our review focuses on the anatomical structure associated with vertebral causes of LBP, which is disc degeneration that can give rise to pain (term discogenic back pain). Here, we summarize the anatomy of IVD, and the pathophysiology of disc degeneration associated with discogenic back pain. We also highlight the key mechanisms underlying disc degeneration that contribute to discogenic back pain and their pathways, current clinical treatments, clinical perspective and directions of future treatments.

## 2. Anatomy of Intervertebral Disc

**Figure 1:**
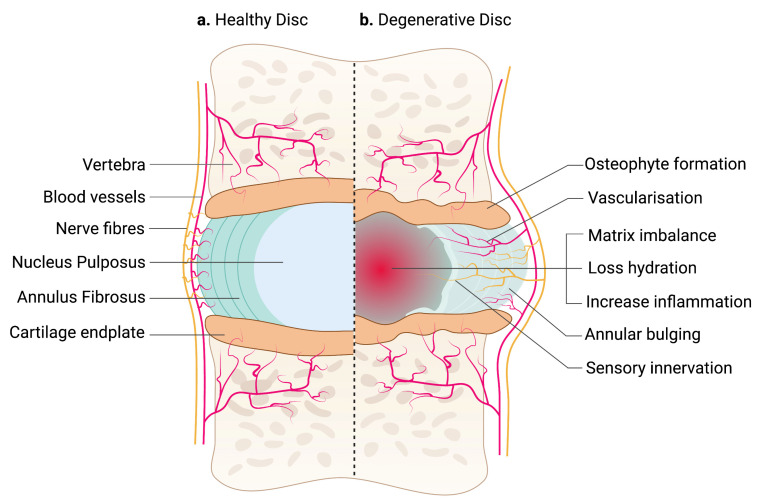
An illustration of a healthy and degenerative IVD. (**a**) IVD comprises two parts: an outer fibrous AF and a center gelatinous NP, which is confined by hyaline CEP. Arteries supply the outermost region of AF, while sinuvertebral nerves innervate the outer third of the AF. NP is avascular and aneural. (**b**) Degenerative IVD is characterized by matrix imbalance, dehydration and inflammation. Blood vessels and nerve fibers are also presented in the inner region of AF and NP. Structural changes in the IVD, including annular bulging and osteophyte formation in the CEP, affecting tissue biomechanics. The schematic was created with BioRender.com.

The IVD between adjacent vertebrae are secondary cartilaginous joints (symphyses) [18]. It unites adjacent vertebrae into a continuous semirigid column important for movement and distributing load [19]. Each IVD consists of an outer fibrous AF and a central gelatinous NP. The nucleus is sandwiched between the hyaline CEP of the vertebrae [20] (Figure 1a).

### 2.1. Annulus Fibrosus

The AF is a fibrous ring consisting of highly organized 15 to 25 concentric layers of collagen fibers called lamellae that surround the NP. Each lamella is formed by strong collagen fibers running obliquely, approximately 30° from one vertebra to another [18]. The fibers of adjacent lamellae cross each other obliquely in opposite directions at angles of more than 60°. Such arrangement allows limited rotation and bending between adjacent vertebrae and enables the IVD to withstand circumferential loads [18,21]. The AF contains 65 to 70% water, and its dry weight contains approximately 20% proteoglycan, 50 to 70% collagen and 2% elastin 8. The thickness of each lamella ranges from 100 to 500 µm, in which the outer lamellae are thicker than the inner lamellae [18,22]. Additionally, each lamella is separated by an interlamellar tissue containing a proteoglycan-rich matrix, elastic fibers, and cells [23]. The AF can be further divided into two zones: the inner AF contains primarily type II collagen fibers produced by rounded chondrocyte-like cells. In contrast, the outer AF contains primarily type I collagen fibers produced by elongated, fusiform, fibroblast-like cells of mesenchymal origin [21,23,24].

### 2.2. Nucleus Pulposus

The NP is the core of the IVD. It contains from 70 to 90% water, while its dry weight contains approximately 35 to 65% proteoglycan, 5 to 20% fine type II collagen fibrils, and the remaining dry weight contains some non-collagenous proteins and elastin [19]. The high-water content of the NP is preserved by proteoglycan and distributes the hydraulic pressure to the ECM to relieve the stress of the IVD [25,26]. The cellular content of the NP consists of a mixture of large notochordal cells and smaller chondrocyte-like mesenchymal cells [27]. The larger and highly vacuolated notochordal cells undergo morphological and functional shifts towards smaller fibrochondrocyte-like cells as the IVD mature [27,28]. Additionally, Mohanty et al. [29] demonstrated that this differentiation is also associated with reduced expression of the key developmental molecule, sonic hedgehog (SHH) and its target Brachyury, which is crucial for the maintenance of NP cells. Because the lamellae of the AF are thinner in the posterior aspect compared to those in the anterior and lateral parts of the IVD, the NP is located between the center and posterior aspects of the IVD [18].

### 2.3. Cartilage End Plate

The CEP is thin layers of hyaline cartilage (approximately 0.6 mm thick) that bind the IVD inferiorly and superiorly to the adjacent bony vertebral bodies [30]. It functions as a mechanical barrier between the NP and vertebra and a gateway for nutrient transport into the IVD from adjacent blood vessels [30]. They are bonded weakly to the underlying vertebral body, making the end plate vulnerable to segmental separation when excessive horizontal stresses are applied [31]. The CEP comprises approximately 60% water, and major dry-weight constituents are collagen type II and proteoglycan [19]. Morphologically, the CEP contained elongated cells parallel to the IVD in alignment with collagen fibers, and the cells produced a collagen-rich interterritorial matrix and a proteoglycan-rich territorial matrix [32].

### 2.4. Blood and Nerve Supplies

The IVD is one of the avascular tissues which only received small arteries supplying the outermost peripheral fibers of the AF [33]. Therefore, the remaining IVD components depend on their delivery of nutrients and oxygen and the removal of waste from the vertebral bone beneath adjacent CEP and from the peripheral AF [34]. Numerous pathological changes, particularly to the CEP, such as bony sclerosis, alterations in blood flow or endplate calcification, may affect transport from the blood vessels through the CEP, which subsequently leads to cell death in the NP, the most affected IVD component due to the cells being located farthest away from supply sources [34]. In general, only the outer third of the AF is innervated by sinuvertebral nerves (SVN), formed by the union of a somatic root from the ventral ramus and an autonomic root provided by the grey ramus [33]. The NP has no nerve supply [35].

## 3. Pathophysiology of Intervertebral Disc Degeneration

Degeneration of the IVD causes dysfunction and instability in the lumbar spine. This is followed by a phase of lumbar spine stabilization due to the formation of osteophytes and disc height narrowing [36]. Both biological and biomechanical factors regulate the IVD degenerative cascade. Biochemical processes play a crucial role in the pathophysiology of the degenerative process and the pain-signaling pathways that cause the clinical features of the disease [37].

The pathologic features of IVD degeneration are illustrated in Figure 1b. The IVD undergoes degenerative changes as early as the first decade of life. Reduction of large vacuolated notochordal cells in the NP is considered the initiation process of disc degeneration. Evidence of reduced progenitor markers such as Tie2+ suggests that there is low capacity for intrinsic tissue regeneration in the IVD [11]. Notochordal markers such as Brachyury have also been associated with glycosaminoglycan deposition and decreased inflammatory mediators’ interleukin (IL)-1β, IL-6, and nerve growth factor (NGF) [38]. Lower cell numbers can alter cell functions in the NP during disc degeneration, resulting in an imbalance between ECM synthesis and degradation [39]. Proteoglycans such as hyaluronan (also known as hyaluronic acid or HA), type II collagen, glycoproteins, and various combinations of elastic fibers are among the ECM components abundantly found in the IVD [40]. A significant decrease in aggrecan leads to loss of proteoglycan and tissue hydration that also causes glycosaminoglycans to be lost, resulting in a reduction in the osmotic pressure of the IVD matrix [41]. Reducing type II collagen synthesis and increasing type I collagen synthesis occurs with a transition to fibrillated tissue quality, and an increase in matrix-degrading enzyme activity, resulting in reduced elasticity and mechanical integrity of the disc. The load-bearing function of the disc is also changed due to reducing hydration [42]. In the AF, degenerative changes are indicated by the delamination of the lamellae and an increased incidence of radial fissures. Degenerative discs have reduced disc height and abnormal mechanical response to loads [43]. Intracellular signaling pathways such as WNT/b-catenin are thought to be involved in the molecular event of disc degeneration [44]. In NP, transforming growth signaling molecules such as bone morphogenetic proteins (BMP6, BMP2), inhibin alpha (INHA), transforming factor alpha (TGFA) and inhibin beta A (INHBA) have been associated with ECM synthesis [45]. Other significant molecules implicated in mediating ECM synthesis in AF include growth factors such as platelet-derived growth factor beta (PDGFB), vascular endothelial growth factor C (VEGFC) and fibroblast growth factor 9 (FGF9), and multiple signaling proteins such as the neurogenic locus notch homolog protein (NOTCH) and WNT [45]. Collectively, these cellular and molecular alterations result in an imbalance of ECM homeostasis, dehydration, reduced mechanical properties and a decrease in the load-bearing capacity of the IVD. Often, there will be little to no pain associated with the changes in IVD.

As disc degeneration progresses with the depletion of proteoglycan, large uncharged molecules such as pro-inflammatory cytokines, serum proteins and neurogenic mediators enter the disc that may trigger inflammation [46]. Both NP and AF cells, as well as macrophages, T cells and neutrophils secrete higher levels of pro-inflammatory cytokines such as tumor necrosis factor (TNF)-α, interferon-γ (IFN-γ), IL-1β, IL-10, IL-4, IL-6, IL-17, IL-2, IL-8, and chemokines such as C-C chemokine receptor 6 (CCR6) and C-C chemokine ligand 20 (CCL20) that is known to mediate inflammation-induced disc degeneration [47,48,49]. Inflammation is also known to mediate ECM degradation. For example, IL-1β and TNF-α have been reported to stimulate matrix degradative enzymes, including A disintegrin and metalloproteinase with thrombospondin motifs (ADAMTS)-4 and ADAMTS-5, metalloproteinase (MMP-2, MMP-1, MMP-13, MMP-3 and MMP-14). In contrast, ECM enzyme inhibitors of metalloproteinases (TIMP)-1, TIMP-3 and TIMP-2 were reduced [50]. This degenerative event causes downregulation of ECM anabolic expression of aggrecan, collagen and SRY-Box Transcription Factor (SOX)-6 [51]. IL-1β-induced expression of MMP-3 through nuclear factor-kappa B (NF-kB), mitogen-activated protein kinase (MAPK) and syndecan 4 results in inflammation-mediated disc degeneration [52]. Our recent study also showed dysregulated glycosylation, particularly higher expression of sialylated and fucosylated N-glycosylation motifs in grade V human degenerative discs that contribute to inflammation and the progression of disc degeneration [53]. IL-17 has also been shown to mediate the inflammatory response via JNK/c-Jun and p38/c-Fos activation in an AP-1-dependent manner in the human NP [54]. At this stage, the discs become dehydrated and reduce in height, and the vertebrae begin to develop osteophytes in response to increasing pressure loads. Calcification of CEP begins as well. Endplate permeability is reduced, resulting in a reduction in metabolic exchange. In the NP, type I collagen crosslinks and creates denser tissue, obstructing the exchange of nutrients and metabolic waste even more [54]. As a result of all these changes, mobility is reduced, and symptoms have often appeared.

**Figure 2:**
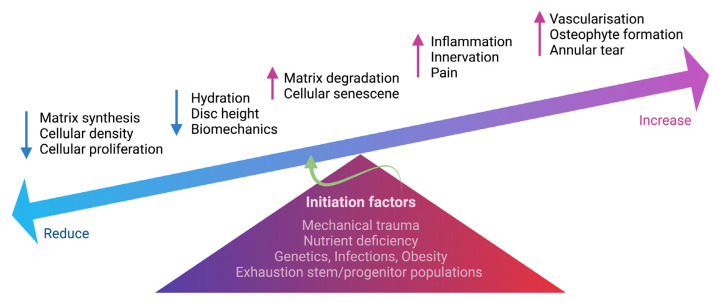
Schematic summarizing the degenerative events in IVD. Multifactorial conditions initiate degenerative processes in the IVD, including mechanical trauma, nutrient deficiency, genetic predisposition, infections, obesity and exhaustion of stem and progenitor cells. The change in IVD cellularity, including cell density, proliferation and cell senescence, can cause an imbalance of ECM homeostasis. Increased matrix degradation and reduced matrix synthesis result in reduced hydration and disc height. Increased inflammation also causes the ingrowth of nerve fibers into the IVD, contributing to discogenic pain. Vascularization, annular tear and osteophyte formation are also present during IVD degeneration. The schematic was created with BioRender.com.

At an advanced stage of disc degeneration, a decrease in joint space causes severe loss of mobility. As a consequence of structural changes, the disc loses its biomechanical function. Fissures occur in the AF, causing the NP to extrude and enable sensory nerve ingrowth and vascularization in the inner AF and NP, contributing to discogenic back pain [55]. Bone spurs continue to grow and cause the spinal canal to narrow, putting pressure on the spinal cord or nerve roots. In the absence of herniation, inflammation and sensory nerve ingrowth have been proposed as a mechanism through which disc cell-secreted cytokines can cause degeneration [56]. Nerve ingrowth in the IVD has been observed to be greater in painful discs than in non-painful discs. Cytokines released by disc cells to draw in immune cells, and those produced by invading immune cells to stimulate disc cells interact intricately. An increase in inflammation enhanced neovascularization around the extruded NP via the vascular endothelial growth factor (VEGF) [57]. Both disc and immune cells continue to produce cytokines such as IL-1 and TNF-α, which can induce the expression of neurotrophins such as NGF and brain-derived neurotrophic factor (BDNF) as a result of nerve ingrowth into aneural discs [58,59,60]. Freemont et al. first reported evidence of nerve ingrowth in painful degenerative discs with higher expression of markers for a protein expressed during axonogenesis, such as growth-associated protein 43 (GAP43), as well as nociceptive neurotransmitters such as substance P [61]. The nociceptive nerve fibers project into the inner third of the AF and into the NP in degenerative discs of chronic LBP patients, indicating an important role for nerve ingrowth into the IVD in the pathogenesis of chronic LBP [61]. The expression of NGF was observed in painful discs with evidence of non-myelinated nerves actively growing (i.e., expressing GAP-43) into the inner AF and NP, suggesting that nerve ingrowth is being driven by local NGF production [62]. Coppes et al. also discovered that painful degenerative discs expressed nociceptive nerve fibers with higher expression of substance P in the outer region of AF, indicating the disc morphologic changes of the discogenic pain [63]. Altogether, disc degeneration associated with discogenic back pain appears to be inflammatory in nature, involving interaction between disc cells and immune cells to produce inflammatory cytokines inducing the release of neurogenic mediators to induce nerve ingrowth into aneural IVD in sensitizing nociception in the painful degenerative discs. Late-stage disc degeneration will almost certainly affect normal activities in LBP patients. We summarize the degenerative hallmarks of IVD in Figure 2.

Morphologically, Thompson et al. first established the grading scheme for the gross changes of midsagittal sections of human lumbar discs. Grade I: the bulging gel of NP, discrete AF lamellae, uniformly thick hyaline of CEP and margin rounded in the vertebral body. Grade II: white fibrous tissue peripherally in NP, the presence of mucinous materials between AF lamellae, irregular CEP thickness and margins pointed in the vertebral body. Grade III: consolidated fibrous tissue observed in the NP, extensive infiltration of mucinous and loss of AF and NP demarcation, focal defects in CEP and early chondro- or osteophytes at vertebral body margins. Grade IV: horizontal clefts parallel to CEP in NP, focal disruptions in AF, fibrocartilage extending from subchondral bone, irregular and focal sclerosis in subchondral bone, and osteophytes smaller than 2 mm in the vertebral body. Grade V: clefts extend through the NP and AF, diffuse sclerosis in CEP and osteophytes larger than 2 mm in the vertebral body [64]. Then, histological classification of IVD degeneration was introduced to determine histological changes on CEP, AF, the boundary of AF and NP, cellularity of NP and matrix of NP [65]. Recently, the histology scoring system was standardized by Le Maitre et al. [66], comprehensively focused on the taxonomy of grading for NP, AF and CEP features, including cellularity, lesions and ECM structure. The NP features include (a) cellularity: single cells in lacunae, cell clusters, necrosis, apoptosis, cell shrinkage, senescence, and acellularity; (b) lesions: tears, clefts and voids; (c) ECM structure: loss of proteoglycan, mucoid degeneration, fibrosis, demarcation of NP/AF and presence of AF and CEP in the NP. AF features include (a) cellularity: acellularity, cell death, change in the OAF cells from elongated to more rounded, and neovascularization; (b) lesions: across and between lamella, enthesis disruption, disruption of bone/AF interface and avulsions; (c) ECM structure: AF bulging, loss of lamella structure, scarring, fibrosis, matrix disorganization, and demarcation with the presence of NP/CEP tissue in the AF [66]. Clinically, spinal magnetic resonance imaging (MRI) has been used for the diagnosis and classification of IVD degeneration. The *Pfirrmann* grading system utilizes T2-weighted MRI and categozises pathologic features into five grades (grade I to grade V) based on structural homogeneity, the distinction of AF and NP, signal intensity and disc height [67].

## 4. Discogenic Low Back Pain

**Figure 3:**
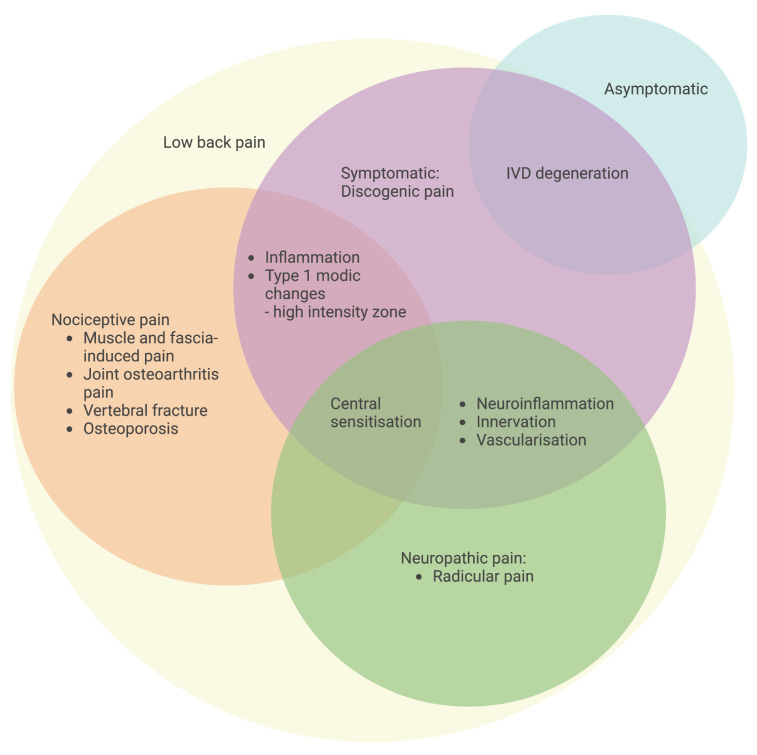
Classification of specific LBP with an emphasis on pain origin. Symptomatic IVD degeneration with discogenic LBP is characterized by inflammation, a high-intensity zone, neuroinflammation-induced innervation and vascularization, and central sensitization for the development of pain. The schematic was created with BioRender.com.

In general, an unpleasant sensory and emotional experience connected with existing or potential tissue damage, or characterized in terms of such damage, is referred to as pain. According to Global Burden of Disease studies, LBP is defined as “pain in the area on the posterior aspect of the body from the lower margin of the twelfth ribs to the lower gluteal folds with or without pain referred into one or both lower limbs that lasts for at least one day” [2]. LBP can be categorized based on where it originates, including radicular LBP, discogenic LBP, facet joint osteoarthritis back pain, muscle and fascia-induced back pain and spontaneous LBP (Figure 3). Radicular pain is caused by compression of one or more nerve roots as a result of stenosis, which is caused by a decrease in disc height or an unstable motion segment. Discogenic LBP is a distinct category of back pain originating from the disc, with MRI findings indicating structural alterations in the discs at lumbar levels and mainly consisting of nociceptive and neuropathic pain [68]. The degeneration of IVD is thought to be one of the contributing reasons associated with nociceptive-type discogenic LBP; however, the pathophysiology of discogenic LBP is not entirely understood [69]. Patients with disc degeneration are two to three times more likely than those who do not have a degenerative disc to experience back pain [70]. Meanwhile, patients with continuous multi-level disc degeneration are more likely to have LBP and have it more severe than those with skipped-level IVD degeneration [71].

Discogenic pain is caused by multifactorial changes in the IVD at a late stage of degeneration that links with the peripheral and central nervous systems. Degenerative features of the IVD at a late stage include loss of disc height, formation of osteophyte, internuclear calcification, and endplate sclerosis as indicated by plain radiographs, as well as reduced hydration as shown by reduced signals of T2-weighted MRI, loss of AF/NP demarcation, irregular cartilage layer, and loss of horizontal trabeculae as indicated by MRI [69]. Increased severity of disc degeneration, in particular neurogenic inflammation-induced ingrowth of sensory nerve fibers (hyperinnervation) along with sensitization of sensory nociceptive processing at peripheral terminals, spinal and supraspinal levels are hypothesized to play a pivotal role in the development of discogenic back pain in disc degeneration [13,70].

The presence of nerve fibers growing within the fissures of AF and even projecting into the deep NP of the disc, in which SVN and basivertebral nerve (BVS) are two nerve fibers that are associated with painful disc degeneration [72]. From peripheral terminals, the primary afferent pain pathways involve the L2 spinal nerve root via sympathetic afferents from the SVN, which innervate the discs. It is not at the same level in the spinal nerves, which explains why many patients with disc herniation complain of sciatica without LBP. The spinal nerve roots are being compressed proximal to the branching site of the SVN [73].

## 5. Mechanisms of Intervertebral Disc Degeneration Underlying Discogenic Low Back Pain

One direct mechanism of IVD degeneration underlying discogenic LBP is nerve ingrowth into the aneural degenerative disc precipitated by inflammatory insults, inducing nociception [62].

### 5.1. Neuroinflammation-Induced Innervation of Nociceptive Fibers in the Discs

Pro-inflammatory cytokines such as IL-1β have been shown to induce the expression of neurotrophins such as NGF and BDNF that result in an increase of innervation, indicated by higher expression of a neuronal marker protein gene product (PGP) 9.5, causing painful degenerated discs [74]. Increased production of NGF and its receptor, tropomyosin receptor kinase A (TrkA), has been demonstrated to cause the ingrowth of the nociceptive nerve into a painful IVD [59]. This evidence points to the presence of a peptidergic population of sensory fibers in the disc that produces neuropeptides, substance P, calcitonin gene-related peptide (CGRP) as well as the TrkA receptor that reacts to NGF [75]. Nerve fibers were discovered across the outer and inner AF that had no accompanying blood vessels and were most likely sensory in nature, typically referred to as free nerve endings that run both obliquely and parallel to the circumferential lamellae of the AF. Neural projections into the inner AF were more common in painful IVD, inside fissures or damaged AF areas [36].

These nociceptive nerve fibers contain neuropeptides derived from the DRG and are classified as small-diameter NGF-sensitive neurons [76], which play a key role in inflammation-induced hyperalgesia [77]. Sensory nerve fibers were also shown to express calcitonin gene-related peptide (CGRP) and nociceptive neurotransmitters such as substance P [78]. To confirm the evidence of sensory nerve fibers, growth-associated protein 43 (GAP43) protein was shown in the inner third of the AF and inner core NP tissue of individuals with persistent back pain associated with degenerative disc [68]. Hyperinnervation of sensory fibers is reported to be stimulated by the interaction of neurogenic mediators, i.e., pro-inflammatory cytokines, neurotrophins and their receptors [79]. In contrast, semaphorin 3A (sema3A), an axonal guidance molecule more abundant in healthy discs than in degenerative discs, may have a repulsive effect on attenuating neuronal ingrowth into healthy discs [80] which is consistent with findings showing that notochordal-rich NP tissue has higher expression of sema3A and chondroitin sulphate, which inhibits neural ingrowth while simultaneously promoting proteoglycan deposition [81].

### 5.2. Neuroinflammation-Induced Nociception

Besides survival and ingrowth of the sensory nerve fibers into the degenerated disc, neurotrophins are also essential for nociceptive processing [82]. NGF activates the p38 MAPK pathway [83] for nociceptive processing by binding to its high-affinity receptor, Trk A, which sensitizes nerve action potential by amplifying the membrane current potential carried by the nonselective ion channel TRPV1 [84,85]. In addition, NGF promotes the production of nociceptive neuropeptides such as substance P and CGRP [86] and central pain neuromodulators like BDNF [87] in the afferent neurons. Anterograde transport of these neurotrophins and neuropeptides to peripheral terminals causes substantial neurogenic inflammation in human degenerated disc cells [80,88]. They are also retrogradely transported to the central terminals in the dorsal horn to enhance central sensitization for nociceptive processing [89,90].

Similarly to TRPV1, NGF also maintains the baseline expression of the acid-sensing ion channel (ASIC) 3 in disc cells via the low-affinity neurotrophin receptor (p75NTR) and ECM-regulated kinase (ERK) signaling [91]. Increased production of lactic acid and protons during anaerobic metabolism causes an increased acidic microenvironment in the disc, which can activate nociceptor ASIC, resulting in musculoskeletal pain [76]. NGF regulates other receptors, such as calcium and potassium voltage-gated ion channels, P2 × 3, and G-protein-coupled receptors [92]. Depolarization of the nociceptor ion channels in the peripheral region of the disc may play a key role in sensitizing nociception to promote discogenic pain and enhance neurogenic inflammation-mediated disc degeneration.

## 6. Pain Pathway

**Figure 4:**
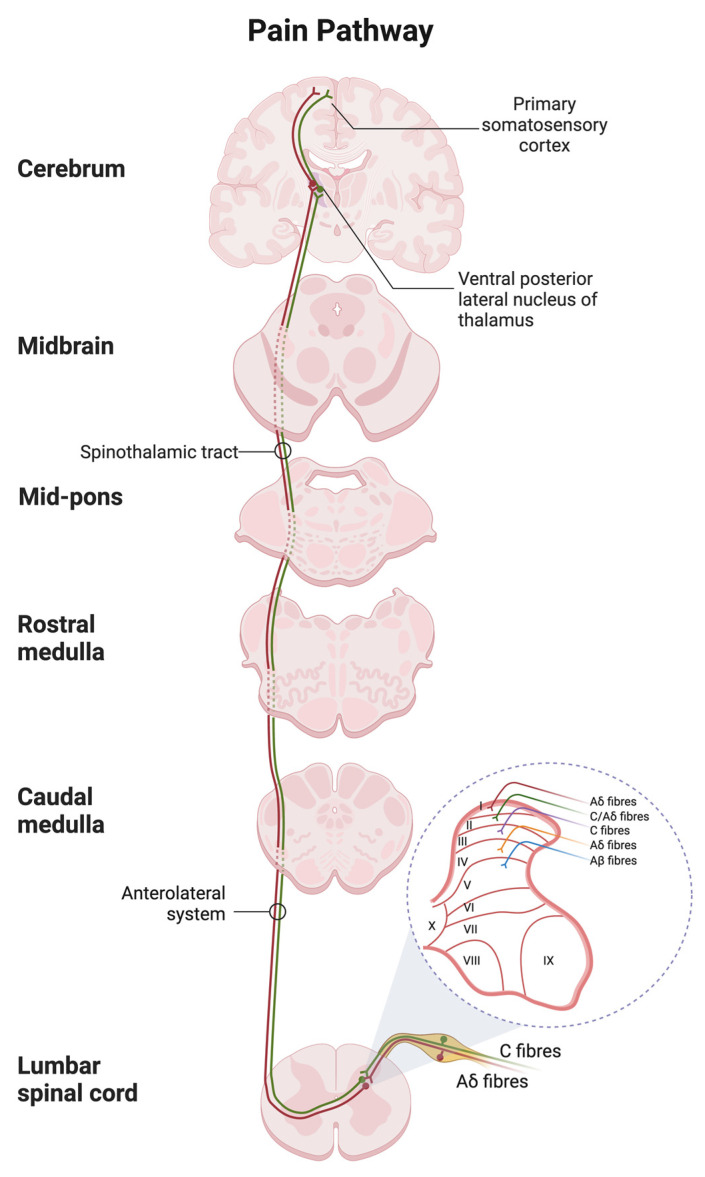
Spinal and supraspinal pathways of pain with the transverse section of the spinal cord showing the origin of the main ascending sensory. Primary afferent neurons transmit the nociceptive impulses via the fast Ad fibers (red) and slow C-type fibers (green) to the dorsal horn of the spinal cord with the laminar organization. The nociceptive input is then relayed to the thalamus and terminated in the postcentral gyrus of the cortex. The schematic was created with BioRender.com.

The nociceptive transmission involves peripheral sensitization, in which the receptors convert the painful or noxious input into an electrical signal. It will be transmitted from peripheral tissue to the central nervous system through ascending (afferent) and descending (efferent) tracts for pain processing [93] (Figure 4).

### 6.1. Peripheral Sensitisation

Depolarization of the nociceptor ion channels in the peripheral region of the disc may play a key role in sensitizing nociception to promote discogenic pain and enhance neurogenic inflammation-mediated disc degeneration. Mechanoreceptors were found in the outer AF of painful discs that were classified as Golgi-type (proprioceptive), Pacini-type (high-frequency vibration or pressure) or Ruffini-type (low-frequency vibration or pressure). In NP there are free nerve endings, Aδ-fibers, C-fibers, Ruffini corpuscles or perivascular fibers [36]. High-threshold mechanoreceptors and polymodal nociceptors are two types of nociceptors that respond to mechanical stimuli and tissue damage mediators, respectively. These nociceptors are activated and sensitized by inflammatory mediators, including cytokines, 5-hydroxytryptamine (5-HT), hydrogen ions (protons), bradykinin, prostaglandins, histamine and leukotrienes to transmit nociceptive information [93].

Depending on the stratification of afferent subtypes, afferent neurons through sympathetic chains project to various sections of the laminae (referred to as Rexed laminae) in the dorsal horn of the spinal cord to convey nociception. The afferent Aδ and C fibers project to lamina I and much of lamina II, which respond to noxious stimulation that is nociceptive specific [94]. Medium-diameter myelinated Aδ afferents convey acute, well-localized “first” or fast pain, while small-diameter unmyelinated C fibers mediate poorly localized, “second” or slow pain [62]. Neurons in laminae III and IV respond predominantly to innocuous stimulation, such as mechanical stimulation through Aβ fibers, but neurons in lamina V receive convergent non-noxious and noxious input via direct (monosynaptic) Aδ and Aβ inputs and indirect (polysynaptic) C fiber inputs [94].

### 6.2. Central Sensitisation

Afferent neurons in the dorsal horn become more excitable during inflammation by amplifying all sensory inputs, increasing the sensitization of Aδ and C fibers in the peripheral terminals at the site of inflammation. The phenotype of Aβ fibers changes into a subpopulation like C fibers, which increases postsynaptic transmission and exaggerates the central response to innocuous stimuli [89]. An increase of presynaptic neurotransmitters sufficiently depolarizes postsynaptic neurons. This includes neurotransmitters such as excitatory amino acids (EAAs), including glutamate and aspartate, which act at both metabotropic (mGlu) receptors and ionotropic receptors such as α-amino-3-hydroxy-5-methyl-4-isoxazole propionic acid receptor (AMPA) and N-methyl-D-aspartate (NMDA); pro-nociceptive tachykinins including substance P and neurokinin (NK) which acts at NK1 and NK2; and pronociceptive neuropeptides, including CGRP action via receptor CGRP1 and CGRP2, to generate receptor excitatory postsynaptic currents in postsynaptic neurons of the dorsal horn [95,96].

The primary activation of AMPA receptors causes rapid depolarization and excitatory transmission, followed by slower and persistent EPSCs mediated by NMDA, mGlu, NK and CGRP receptors [95]. Furthermore, neurotrophins such as BDNF enhance glutamatergic transmission, which is found in the lamina I and lamina II of the dorsal horn. BDNF binds to its high-affinity receptor of Trk B at presynaptic neurons to modulate glutamate and neuropeptide release. At the postsynaptic membrane, BDNF activates Trk B by autophosphorylation, leading to the activation of MEK/ERK and PLC/PKC cascades. The second messengers of these pathways phosphorylate NMDA, and AMPA receptors modulate their activity. NMDA receptors are implicated in BDNF-induced AMPA receptor potentiation by regulating receptor phosphorylation and trafficking [97]. In addition, synaptic transmission induces the expression of an immediate early gene c-Fos messenger system in postsynaptic neurons to convey nociceptive input to higher centers in the brain [98].

### 6.3. Ascending and Descending Tracts

Afferent neurons convey nociceptive signals to the brain by crossing over to the opposite side (contralateral), relay pain signals along the spinothalamic tract to the thalamus and terminate in the somatosensory cortices, insula, anterior cingulate cortex, prefrontal cortex and periaqueductal grey matter (PAG) for pain localization. Pain signals are also conducted through the spinoreticulothalamic tract to the reticular formation of the brainstem before projecting to the thalamus and hypothalamus for emotional pain processing [93]. Furthermore, the ascending pain pathway can be modulated by the activity of second-order neurons of the descending pain pathway that release neurotransmitters, resulting in facilitation (potentiate) or inhibition (suppress). Descending tracts originate from the hypothalamus, limbic areas or cortex and then project to the PAG and brainstem to terminate in the dorsal horn of the spinal cord [99].

## 7. Current Treatments

Patients with degenerative disc disease, especially in the lumbosacral region, generally present with mechanical LBP, especially with loading (e.g., prolonged standing/sitting, carrying a heavy load). Prolapsed disc material, especially the NP, can cause an inflammatory response and irritation to the nerve root. The physical narrowing of the degenerative process can lead to symptoms and signs of neurological disturbance such as neurogenic claudication, radiculopathy and, the worst of the spectrum, cauda equina syndrome. The aim of treatment in patients with LBP without neurological deficits is pain management. There is a plethora of conservative treatment options for most patients with LBP; however, no evidence exists to prove which one is preferable to others. Patients who have prolonged symptoms and have failed conservative therapy are advised to have operative treatment, either decompression surgery with or without fusion [100].

Conservative management of LBP is generally divided into pharmacological and nonpharmacological treatment. Nonsteroidal anti-inflammatory drugs (NSAIDs) are the mainstay of pharmacological agents used to treat pain symptoms by reducing the inflammatory component of the pain pathways. Cyclooxygenase-2 (COX-2) inhibitors are a type of NSAIDs that specifically blocks COX-2 enzymes, thus relieving the inflammation and pain with less adverse gastrointestinal effects than nonselective NSAIDs. NSAIDs can be combined with other analgesic medications like paracetamol and weak opioids (i.e., tramadol), aiming to target the pain pathway at different levels. Other pharmacological treatments include neuropathic pain medications such as anticonvulsants and/or antidepressants (i.e., gabapentin, pregabalin and duloxetine). Topical or patch forms of analgesia are effective, but there is no evidence of a long-term benefit. The role of muscle relaxants is still conflicting, but we prefer using them in the acute phase and short duration to relieve the muscle spasms in the acute phase.

Nonpharmacological treatment mainly involves a rehabilitation program which aims to return patients to their regular functional activity (i.e., improve range of movement) and prevent further injury. This can be achieved by optimization of core muscle strength, endurance and coordination. Back schools are educational and training programs with lessons given to patients or workers by a therapist aiming at treating or preventing LBP [101]. They are a commonly used nonpharmacological intervention, especially within the occupational health setting. Overall, the evidence for using back schools to treat chronic low back pain is equivocal. Current modalities of multimodal rehabilitation mainly consist of exercise therapy combined with cognitive behavioral training and are more effective in reducing disability and pain-related fear than exercise therapy alone. An effective rehabilitation treatment will reduce the dependence on pharmacological treatment. As noted, interventional treatment for LBP involves a variety of procedures utilized by surgeons and pain specialists. Peripheral nerve blocks, trigger-point injections, epidural injections, facet joint injections and radiofrequency ablation are all standard procedures [100].

Surgical intervention will be the final step in the algorithm for managing degenerative disc disease. The indication of surgery will be the failure of conservative treatments for at least two to three months. Other indications will be interference or loss of ability to do activities in daily life due to progressive neurogenic claudication, which limits walking due to pain, weakening of the muscles, paresthesia in the buttocks or lower extremities and rapidly progressing nerve impairment. The presence of cauda equina syndrome would redeem an early decompression surgery. In essence, decompression surgery improves outcomes in patients with moderate to severe symptoms of degenerative spinal stenosis, especially with predominant leg symptoms without instability. Decompression with fusion is indicated when substantiated by the presence of instability preoperatively or following a decompression procedure. As life expectancy continues to increase, the primary degenerative sagittal imbalance is diagnosed in an increasing number of older adults. The ultimate spectrum of surgery will aim to correct spinal column deformity (i.e., correction of sagittal imbalance), which might require spinal osteotomy with spinal instrumentation and fusion. Corrective surgery for this sagittal deformity is becoming more common, and the effectiveness of the procedure has shown promising outcomes in selected cohorts [100].

## 8. Clinical Perspective and Future Treatments

LBP is the most common musculoskeletal disorder impacting the quality of life in patients. The most common cause of LBP in the elderly population is degenerative disc disease and its associated pathologies [2]. The treatment strategies depend on the age of presentation, the severity of the degeneration of the disc associated with the neural compression, stability of the functional spinal unit and global balance of the spinal column (i.e., sagittal balance). Wu et al. [102] suggested a treatment ladder for degenerative disc disease: pain alleviation through conservative management, restorative, reconstructive, replacement and, lastly, rigid fusion. Restorative therapy entails molecular approaches, such as gene, growth factor and cell therapy, that aim to restore the anabolic phenotype of the disc and decrease the catabolic function in an attempt to repair disc damage. These approaches are still in the early stages of clinical, experimental laboratory and animal trials, with extensive clinical effectiveness yet to be proven. In addition, precision medicine is an emerging idea in regenerative therapy, particularly personalized biomaterial-based tissue engineering tailored to the severity of disc degeneration [9]. Hence, further clinical studies provide an outlook of their potential and incorporation of their role in the algorithm for the management of degenerative disc diseases.

## 9. Conclusions

The pathogenesis of degenerative disc disease remains complicated and challenging. Understanding the anatomy and pathophysiology of disc degeneration can aid in the selection of the most effective treatment and the management of degenerative discs, which is also helpful in modelling painful disc degeneration to study IVD pathology as well as to identify novel pathways and signaling molecules as a therapeutic target in the treatment of disc-degeneration-associated discogenic pain.

## Data Availability

Not applicable.
